# The Bristol Sponge Microbiome Collection: A Unique Repository of Deep-Sea Microorganisms and Associated Natural Products

**DOI:** 10.3390/antibiotics9080509

**Published:** 2020-08-13

**Authors:** Sam E. Williams, Henry L. Stennett, Catherine R. Back, Kavita Tiwari, Jorge Ojeda Gomez, Martin R. Challand, Katharine R. Hendry, James Spencer, Angela E. Essex-Lopresti, Christine L. Willis, Paul Curnow, Paul R. Race

**Affiliations:** 1 School of Biochemistry, University of Bristol, University Walk, Bristol BS8 1TD, UK; samuel.williams@bristol.ac.uk (S.E.W.); henry.stennett@bristol.ac.uk (H.L.S.); catherine.back@bristol.ac.uk (C.R.B.); kt17533@bristol.ac.uk (K.T.); Martin.Challand@bristol.ac.uk (M.R.C.); 2 BrisSynBio Synthetic Biology Research Centre, Tyndall Avenue, Bristol BS8 1TQ, UK; chris.willis@bristol.ac.uk; 3 Technical Services, Faculty of Life Sciences, University of Bristol, University Walk, Bristol BS8 1TD, UK; Jorge.OjedaGomez@bristol.ac.uk; 4 School of Earth Sciences, University of Bristol, Queens Road, Bristol BS8 1RJ, UK; k.hendry@bristol.ac.uk; 5 School of Cellular and Molecular Medicine, University of Bristol, University Walk, Bristol BS8 1TD, UK; Jim.Spencer@bristol.ac.uk; 6 Defence Science and Technology Laboratory, Porton Down, Salisbury SP4 0JQ, UK; AEELOPRESTI@mail.dstl.gov.uk; 7 School of Chemistry, University of Bristol, Cantock’s Close, Bristol BS8 1TS, UK

**Keywords:** natural products, microbiology, antibiotics, extremophiles, bioprospecting, actinomycetes

## Abstract

The deep ocean is the largest habitat for life on Earth, though the microorganisms that occupy this unique environmental niche remain largely unexplored. Due to the significant logistical and operational challenges associated with accessing the deep ocean, bioprospecting programmes that seek to generate novel products from marine organisms have, to date, focused predominantly on samples recovered from shallow seas. For this reason, the deep ocean remains a largely untapped resource of novel microbiological life and associated natural products. Here we report the establishment of the Bristol Sponge Microbiome Collection (BISECT), a unique repository of deep-sea microorganisms and associated metabolites isolated from the microbiota of marine sponges, recovered from previously unsurveyed regions of the mid Atlantic Ocean, at depths of 0.3–3 km. An integrated biodiscovery pipeline comprising molecular, genetic, bioinformatic and analytical tools is also described, which is being applied to interrogate this collection. The potential of this approach is illustrated using data reporting our initial efforts to identify antimicrobial natural product lead compounds. Prospects for the use of BISECT to address allied pharmaceutical needs, along with mechanisms of access to the collection are also discussed

## 1. Introduction

Natural products and their derivatives are preeminent sources of lead compounds for pharmaceutical development, accounting for >50% of all small molecule-based drugs in current clinical use [1,2]. The influence of natural product chemistry on drug discovery has not materially decreased for more than 30 years, with drug approval statistics over the past decade providing unequivocal evidence that natural product scaffolds remain the ideal starting point for the development of new medicines [3]. Despite this, enthusiasm for natural products within the pharmaceutical industry has progressively waned since the early 1990s, usurped by structure-guided approaches and combinatorial chemistry. Regrettably, the decision to transition away from natural products as a starting point for drug discovery has led to a decline in the productivity of the pharmaceutical industry, with a consensus now emerging that natural product-based discovery approaches were prematurely jettisoned [4,5]. 

One of the major criticisms levelled at natural product-based approaches is the high probability of compound rediscovery [6]. Repeated screening of environmental isolates from geographically-related sampling sites frequently delivers molecules that are too similar to known compounds to make commercial development tractable. A further complication is that many of the microbial gene clusters that encode the biosynthetic machinery required to assemble natural product scaffolds are inactive, repressed or ‘silent’ under standard laboratory culturing conditions [7]. This combination of chemical redundancy and dormant metabolism reduces the likelihood of isolating novel bioactive compounds, even from sizeable culture collections. 

One solution to the problem of rediscovery is to employ a sampling approach that targets previously unexplored environmental niches. By focusing on such environments, the risk of compound rediscovery is significantly reduced [8,9]. Marine ecosystems have long been viewed as an attractive source of novel microorganisms and associated natural products [10]. Marine bioprospecting programmes have in particular targeted sponges (Porifera); ancient metazoans that have existed for ~600 million years. These sessile organisms are known to host a diverse microbiome comprising a plethora of bacteria and fungi, which biosynthesise numerous bioactive compounds, including antimicrobial, antiviral, antifungal and anticancer agents [11,12]. The microbes that make up the sponge microbiome produce these bioactive compounds for their own protection and to compete with other microorganisms for resources, thus potentially contributing to the protection of the sponge against pathogens [13]. As pioneer marine colonizers, sponges are highly evolved and well-suited to survival in extreme environments, including the deep ocean. Consequently, the microbiota housed by the sponge can be subjected to extremes of pressure and temperature, and receive minimal exposure to light. These conditions necessitate the development of metabolic innovations, which in turn translate into novel biosynthetic capabilities and resulting natural product chemistries [14]. 

Historically, bioprospecting programmes targeting sponges and their microbiota have focused on specimens recovered from shallow waters. This is a direct consequence of the operational challenges associated with material recovery from the deep ocean [15]. However, recent progress in the development of unmanned remotely operated vehicles (ROVs), which can be deployed to recover sponge samples from previously inaccessible deep-sea niches, is now unlocking new opportunities for natural product screening programmes targeting these unique biological samples. 

Here we report the establishment of the Bristol Sponge Microbiome Collection (BISECT), a growing collection of marine microorganisms isolated from a unique repository of 75 mid-Atlantic deep-sea sponge tissue samples. Significantly, each sponge sample within the collection was recovered from a previously unsurveyed marine niche using an ROV deployed from the RRS James Cook. Our hope is this unique niche allows BISECT to expand the total microbial diversity and function of similar repositories: such as at the Marine Biodiscovery Center [16] and Fundación MEDINA’s collection [17]. Allied to the development of this collection, we also describe an end-to-end biodiscovery pipeline comprising a suite of in vivo, in vitro and in silico tools, which are being applied to establish the biosynthetic potential of the contents of BISECT. To exemplify our approach, we present data relating to our initial attempts to screen this collection for the presence of antimicrobial natural products. Finally, we outline mechanisms of access to the collection in an effort to nucleate interest and engagement from academic colleagues, and to maximise the impact of BISECT on the research community. 

## 2. Results

### 2.1. Sponge Sample Collection

**Figure 1:**
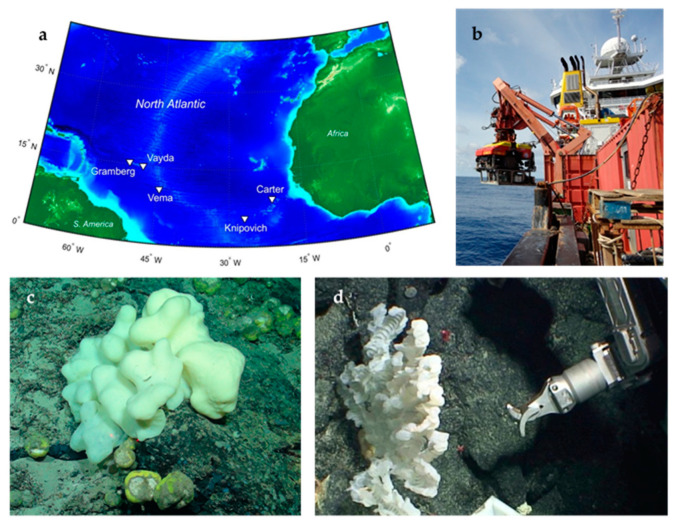
(**a**) Map of the deep-sea sampling areas (starred) from the JC094 research cruise in the mid-Atlantic Ocean, created using ETOPO1 bathymetry. (**b**) Research vessel RSS James Cook and associated remotely operated vehicle (ROV) used for collecting samples. (**c**) One of the sponges collected. (**d**) The robotic arm of the ROV.

**Figure 2:**
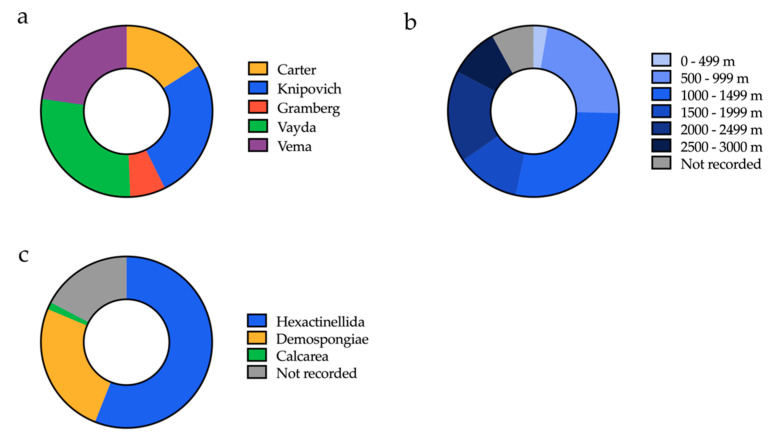
Metadata for 75 sponges within the BISECT samples. (**a**) Number of sponges collected from each sampling site (see Figure 1). (**b**) Depth from which each of these sponges was collected. (**c**) Initial taxonomic assignment of the sponges (estimated based on morphology).

In 2013 the Tracing Ocean Processes Using Corals and Sediments (TROPICS) research cruise (RRS James Cook, expedition JC094) collected a series of marine samples including Porifera (sea sponge) from five different sites across the equatorial Atlantic between Tenerife and Trinidad (Figure 1a and Figure 2a). Sampling was executed on the seabed at depths of between 298 and 2985 m using a remotely operated vehicle (ROV) (Figure 1b), furnished with a robotic arm or suction device (Figure 1d). Two-thirds of the sponges were collected at depths of 1 km or greater (Figure 2b). The properties of the seawater were measured for some sampling sites, and ranged between salinities of 34.86–35.09 PSU, oxygen concentrations of 36.77–236.25 μmol/kg, and temperatures of 2.8–9.9 °C. A range of different marine Porifera were selected from the Demospongiae (common sponge), Calcarea (calcareous sponge) and Hexactinellida (glass sponge) taxa (Figure 2c). Once at the surface, samples were photographed, subsampled and then frozen at −80 °C for the return journey back to Bristol.

### 2.2. Isolation of Strains Producing Active Compounds

#### 2.2.1. Bacterial Culturing and Isolation

Six sponge tissue samples, known by the in-house unofficial designation B01607, B01641, B01661, B0789, B0078 and B01171 (Table 1) were washed in artificial sea water (ASW) in order to reduce the number of non-sponge-associated bacterial cells, then homogenised in a pestle and mortar and spread onto a variety of different agars (Supplementary Table S1) containing ASW. It was understood that that the methods of collecting the sponge samples and culturing the strains was not infallible, and the strains could not be definitely identified as ‘sponge-associated’, but by washing the samples we could increase confidence that the majority were bacteria from the sponge and not contaminants. Isolation protocols were improved successively with each sponge by using a wider range of media each time (Table 2) and deliberately favouring agars known to promote the growth of actinomycetes strains. The agar plates were incubated at 28 °C or 4 °C for 4–10 weeks. Temperature was found to be an important variable with a higher number of isolates being recovered at 28 °C for all sponges, albeit with considerable differences in this ratio between the different sponges. Individual colonies were picked according to their morphology and maintained on agar plates at their isolation temperature for up to four weeks. For long term storage confluent cultures of each isolate were supplemented with glycerol (20% final concentration) and flash frozen in liquid nitrogen prior to archival at −70 °C.

#### 2.2.2. Screening of Isolated Strains for Antibiotic Activity

**Figure 3:**
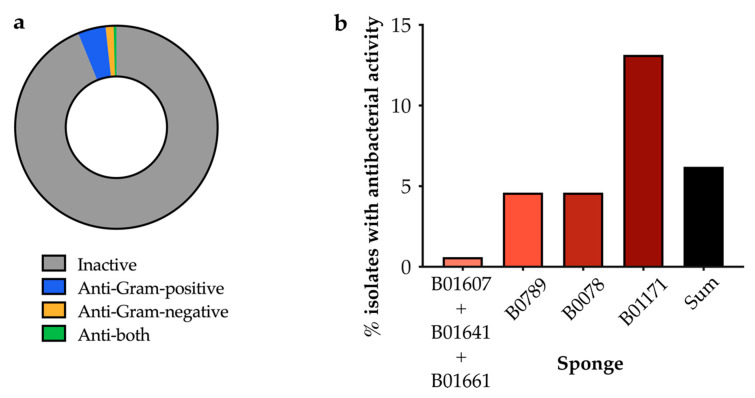
(**a**) The proportion of cultured sponge bacteria exhibiting antibacterial activity (*n* = 699). (**b**) The percentage of isolates with antibacterial activity isolated from sponges B01607 + B01641 + B01661 (total from all three sponges), B0789, B0078 and B01171. *Sum* is the overall percentage considering all samples shown.

Each isolated marine strain was initially screened for antimicrobial activity against a panel of pathogenic bacteria for which new antibiotics are critically needed: *Staphylococcus aureus* Newman and Mu50, *Pseudomonas aeruginosa* PAO1, *Klebsiella pneumoniae* NCTC 5055, *Acinetobacter baumannii* ATCC 19606, and *Escherichia coli* BW25113 (Supplementary Table S2). Various whole colony activity assays were trialed, including the cross-streak method [18], the agar plug diffusion method [19] and the soft agar overlay method (modified from Anand et al., 2006) [20]. In our hands the soft agar overlay assay yielded the most consistent results and allowed high throughput testing of a large number of isolates (data not shown). Figure 3a demonstrates the proportion of sponge isolates that inhibited the growth of pathogens in these soft agar overlay assays. Overall, 6.2% (43/699) of our isolates showed antibacterial activity. Isolates that only showed activity against Gram-positive pathogens were more common than those which showed activity against Gram-negative pathogens (Figure 3a). In an effort to increase the number and variety of isolated strains and ‘hits’, we progressively increased and diversified the isolation media for each different sponge that we processed. This resulted in a larger proportion of isolates that displayed antibacterial activity (Figure 3b). Just 0.6% of isolates from the first three sponges (B01607, B01641 and B01661) had activity (1/166), compared to 4.6% for the next two sponges (B0789 and B0078; 6/131 and 8/174) and 13% for the final sponge (B01171; 28/213). The strains with antimicrobial activity from sponge B01171 are shown in Supplementary Table S3.

### 2.3. Composition of Cultured Bacteria 

#### 2.3.1. Distribution of Isolated Strains from Sponges B01641, B01661, B0789 and B0078

Bacterial strains were selected from the isolation plates based on distinct morphologies, media composition and isolation temperature. Therefore, the bacteria chosen for further analysis are not the entirety of the cultured bacteria, but rather provide a representative overview of unique culturable isolates. Fifty-five strains were isolated from sponge B01641, 73 strains from B01661, 137 strains from sponge B0789 and 182 strains from B0078. 16S rRNA gene sequencing and subsequent BLAST analyses were completed for 28 isolates from B01641 (51%), 27 isolates from B01661 (37%), 47 isolates from B0789 (34%) and 149 isolates from B0078 (82%). Bacteria identified from the genera *Staphylococcus* (52) and *Cutibacterium* (9), along with the species *Pseudomonas aeruginosa* (6) and *Escherichia fergusonii* (5), were excluded from the analysis due to the high likelihood that they represent contamination rather than authentic marine bacteria.

A total of four bacterial phyla were cultured from the sponge samples: Proteobacteria (*n* = 48), Bacteroidetes (*n* = 43), Actinobacteria (*n* = 39) and Firmicutes (*n* = 15). There were notable differences in phyla and genera of strains recovered in sample B0078 compared to the earlier sponges. As listed in Table 2, extracts from B0078 were inoculated on different media compositions, as well as supplemented with additives. After processing sponges B01641, B01661 and B0789 we chose to alter the isolation agars for B0078 to more actinomycetes-selective media types, and to add nalidixic acid to prevent the growth of fast-growing Gram-negative bacteria [21]. We otherwise deliberately avoided actinomycete-enriching pretreatments on our isolation plates because several studies have indicated that these procedures, which were developed for terrestrial actinomycetes, eliminate deep-sea actinomycetes [22,23,24].

**Figure 4:**
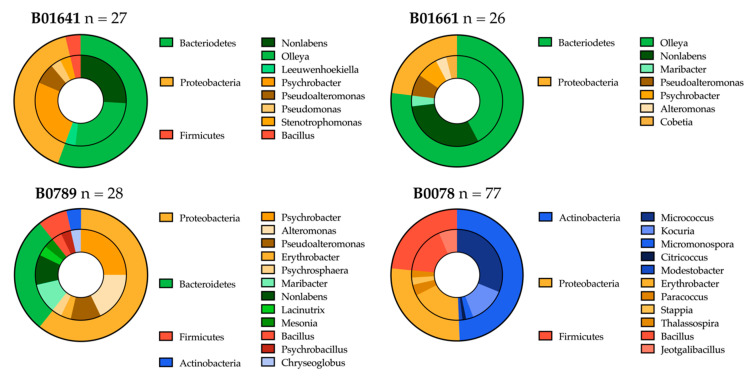
Distribution of bacterial isolates cultured from four deep-sea sponge samples. Molecular taxonomy is based on 16S rRNA gene sequences either at the phylum level (outer circle) or the genus level (inner circle).

These measures proved successful in isolating an increased number of strains of Actinobacteria and a decreased number of Bacteroidetes. Whilst only 1 strain of Actinobacteria was cultured from B01641, B01661 and B0789, 37 Actinobacteria were recovered from B0078. No bacteria from the phyla Bacteroidetes were recovered from sponge sample B0078 (Figure 4). The Actinobacteria primarily consisted of the genus *Micrococcus* (*n* = 24), with the remainder represented by the genera *Kocuria* (*n* = 10), *Micromonospora* (*n* = 2), *Modestobacter* (*n* = 1), *Citricoccus* (*n* = 1) and *Chryseoglobus* (*n* = 1). 

Representatives from 8 different genera were recovered from B01641, 7 from B01661, 11 from B0078 and 12 genera from sponge B0789 (Figure 4). The total diversity of the bacterial isolates was spread across 26 genera. The highest number of genera (*n* = 11) belonged to the phylum Proteobacteria, 6 genera were recovered from Bacteroidetes and Actinobacteria, with only three genera recovered from the phylum Firmicutes. No single genus was isolated from all four sponge samples, but *Psychrobacter, Pseudoalteromonas* and *Nonlabens* were all recovered from the first three sponges (B01641, B01661 and B0789). Considering the higher level of 16S rRNA gene sequence sampling for sponge B0078, bacterial diversity does appear to be higher for B0789, but this is a likely consequence of the use of more Actinobacteria-appropriate media and the inclusion of antibiotics to limit the growth of fast-growing Gram-negative bacteria. 

#### 2.3.2. Phylogenetic Relationship of Cultured Actinobacteria

**Figure 5:**
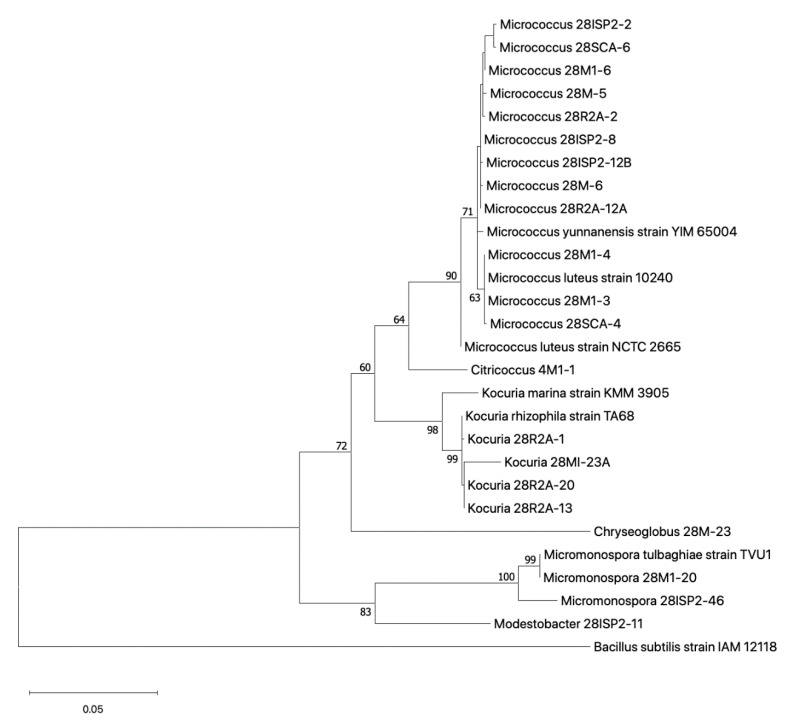
Phylogenetic tree of selected Actinobacteria bacterial strains isolated from deep-sea sponges B0789 and B0078. The trees were constructed by comparison of a ~1400-bp region of the 16S rRNA gene sequence using neighbour-joining analysis with Tamura-Nei model. Bootstrap values expressed as percentages of 1000 replications and branches with less than 60% bootstrap support not displayed. *Bacillus subtilis* IAM 12118 used as an outgroup and tree rooted at the outgroup. Each isolate was allocated genus prefix based on top BLAST result of the 16S rRNA gene database. Taxa with species level names were chosen from NCBI blast database for reference.

A phylogenetic tree was constructed of isolates using partial (approximately 1400 bp) 16S rRNA gene sequences belonging to the Actinobacteria phylum (Figure 5). Twenty-one Actinobacteria isolates are represented in the tree, of which 20 belong to the order Actinomycetales and one to the order Geodermatophilales (Modestobacter 28ISP2-11). Twelve isolates are allocated to the *Micrococcus* genus, four to *Kocuria*, two to *Micromonospora*, one to *Chyrseoglobus*, one to *Citrococcus* and one to *Modestobacter*.

### 2.4. OSMAC Screen of 90 Isolates 

**Figure 6:**
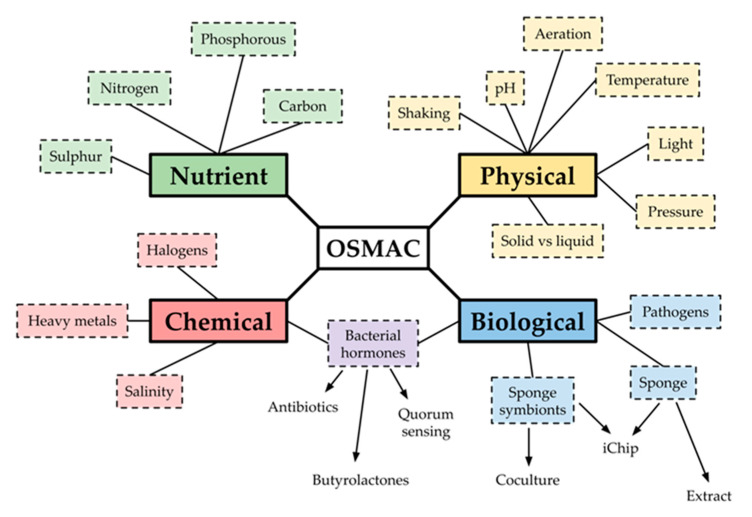
Some of the growth conditions that can be altered in an OSMAC screen targeting deep-sea sponge bacteria.

Only a small fraction of the isolates showed antibacterial activity under their initial isolation conditions (Figure 3). Therefore, we decided to utilise the “One Strain MAny Compounds” (OSMAC) strategy [25] (Figure 6), which varies the cultivation conditions in an effort to force the activation of ‘silent’ gene clusters and, thus, cause the production of bioactive secondary metabolites. This method was used to investigate the activity of 90 isolates from the collection, none of which originally showed antibacterial activity. This selection included 57 unique strains (based on their 16S rRNA gene sequences) and 33 strains that were isolated on the actinomycete-targeting media R2A and M1 (16S rRNA genes were not sequenced). Two OSMAC screens were designed using the 2018 review by Romano et al. [26] as a guide. The first screen altered the composition of ISP2, a complex medium for the isolation of fastidious actinomycetes [27], which had produced the most ‘hits’ during our initial work (Table 2). The second screen used the more chemically defined M9 agar and altered the type and concentration of carbon source present. 

#### 2.4.1. OSMAC Screen Using IPS2 Media

**Figure 7:**
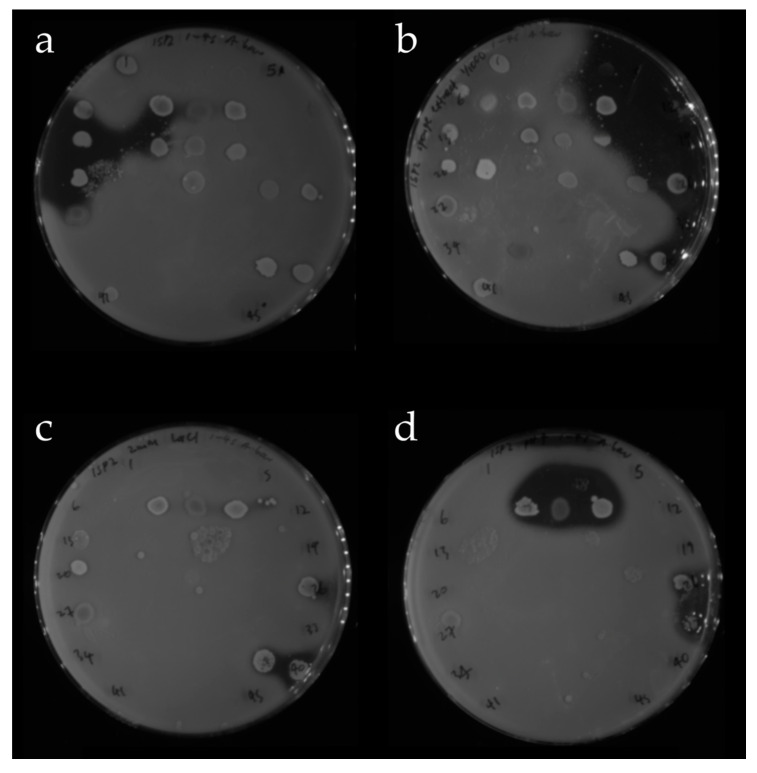
Four OSMAC plates from the ISP2 screen (**a**) ISP2 agar, (**b**) ISP2 agar with sponge extract, (**c**) ISP2 agar with 2 mM LaCl_3_, and (**d**) ISP2 agar prepared at pH 5. Each was spotted with the same 45 sponge bacteria in the same positions, grown for a week at 28 °C and overlaid with a soft agar suspension of *A. baumannii*. The plates are opaque where either or both of the sponge bacteria and *A. baumannii* have grown. Clear zones indicate that the growth of *A. baumannii* was inhibited.

Strains were grown as small patches on agar plates of each OSMAC medium. The plates were overlaid with a Gram-positive (*S. aureus* Newman) and a Gram-negative (*A. baumannii*) pathogen, and visible zones of inhibition indicated that the marine isolates were producing diffusible antibacterial natural products. These clearance zones appeared markedly different on each of the OSMAC plates, suggesting that these different conditions were indeed activating silent biosynthetic gene clusters (Figure 7).

Our rationale for each OSMAC condition for the ISP2 screen is as follows: Firstly, sponge-associated bacteria may only produce antibiotics when stressed, so the OSMAC media accounts for this by encompassing pH changes, hypersalinity (10% *w/v* NaCl compared to 3.5% for seawater [28]), cold, heat and anaerobic shock. Secondly, sponge bacteria might also require certain biochemical signals to produce antibiotics, so the media included sponge extract, NaBr, LaCl_3_, chitin, or γ-butyrolactone [26]. Finally, co-culturing marine bacteria with human pathogens has been shown to upregulate antibiotic production, so we also used a suspension of lysed pathogens in one of the media conditions [29].

**Figure 8:**
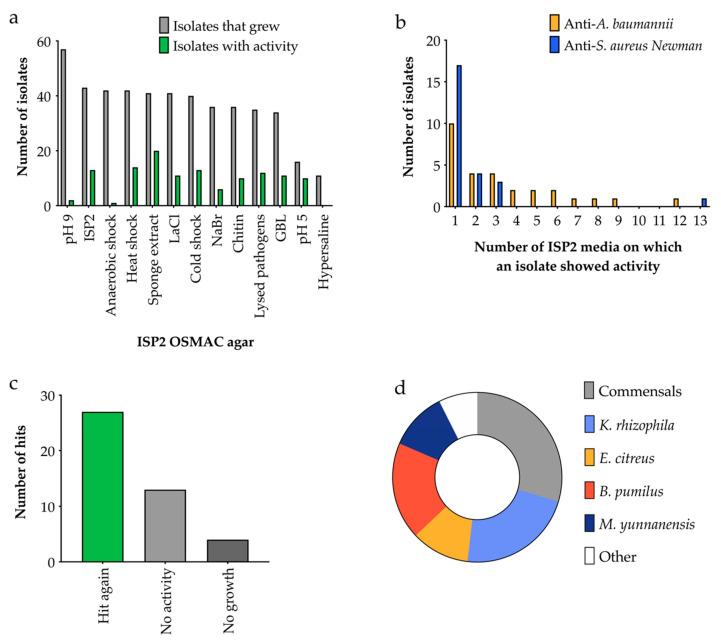
Results of the OSMAC screen of 90 isolates on ISP2 media with different additives. (**a**) Number of isolates displaying antibacterial activity against *A. baumannii* or *S. aureus* Newman, *n* = 90. (**b**) Number of isolates that exhibited antimicrobial activity on different ISP2 media. (**c**) The number of ‘hits’ which retained activity when grown axenically, *n* = 44. (**d**) The closest relatives of the ‘hit again’ isolates based on 16S rRNA gene sequencing, *n* = 27.

The first OSMAC screen used ISP2 as a base and the results shown in Figure 8 indicate that the ability of bacteria to grow on an altered OSMAC agar was not linked to their ability to inhibit pathogen growth. For example, over three times as many isolates grew on ISP2 at pH = 9 compared to pH = 5 (57 versus 16). However, fewer of the pH = 9 isolates inhibited pathogen growth; only 2 isolates versus 10 grown at pH = 5. Intriguingly almost all strains with antimicrobial activity showed activity on only a single medium, implying a specific requirement for that condition rather than a general effect. 

We attempted to corroborate the results of the OSMAC screen by re-culturing and re-screening those strains with antimicrobial activity. The growth inhibition was recapitulated in 61% of the re-cultured strains (Figure 8c). The 16S rRNA genes of the strains with antimicrobial activity were sequenced (Figure 8d). A significant proportion were human commensals (*E. fergusonii, C. acnes*, *S. aureus*), which were assumed to be contaminants rather than sponge symbionts. However, the remainder of the isolates were entirely consistent with the deep-sea origins of the samples [30,31], including multiple samples of *Kocuria rhizophila, Bacillus pumilus, Erythrobacter citreus*, and *Micrococcus yunnanensis* (Figure 8d, Supplementary Table S4). A comprehensive analysis of these bacteria is ongoing.

#### 2.4.2. OSMAC Screen Using M9 Media

The strains used in the ISP2 screen (Section 2.4.1) were screened in parallel with the well-defined M9 media. This allowed variation of the carbon source; different carbon sources can inhibit or stimulate the metabolic pathways that might lead to bioactive secondary metabolites [7,9].

**Figure 9:**
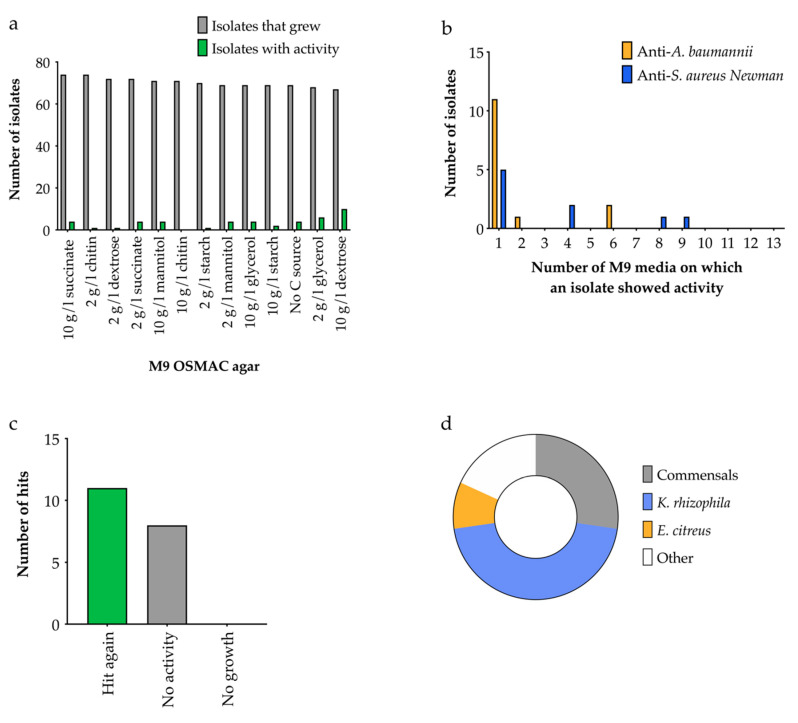
Results of the OSMAC screen on M9 media with different carbon sources. (**a**) Number of isolates displaying antibacterial activity against *A. baumannii* or *S. aureus* Newman, *n* = 90. (**b**) Number of isolates that exhibited antimicrobial activity on different M9 media. (**c**) The number of ‘hits’ which retained activity when grown axenically, *n* = 19. (**d**) The closest relatives of the ‘hit again’ isolates based on 16S rRNA gene sequencing, *n* = 11.

A greater number of bacteria were recovered from OSMAC screens on M9 agar versus ISP2, and this was largely insensitive to the carbon source (Figure 9a). M9 agar with 10 g/L dextrose produced the greatest number of hits (Figure 9a), suggesting that carbon catabolite repression was not significantly inhibiting natural product biosynthesis in these isolates. Again, most ‘hits’ only showed activity on one medium (Figure 9b). Fifty-eight percent of the M9 hits showed activity again when grown axenically (as a single species culture) and of these, three isolates were identified as human commensals (Figure 9c). A significant proportion of the hits were again related to *K. rhizophila* and *E. citreus,* similarly to the ISP2 media screen, but a *Micromonospora* and a *Stappia* species were unique to the M9 screen. Only four of 22 growth-inhibiting strains were active in both screens, demonstrating that using a wide range of conditions for an OSMAC screen is crucial.

## 3. Discussion

Here we report a biodiscovery pipeline for the isolation and study of bacteria associated with sponges harvested from previously inaccessible and unsurveyed areas, deep in the mid-Atlantic Ocean, and the establishment of the Bristol Sponge Microbiome Collection (BISECT) of sponge-associated marine microorganisms. This pipeline attempts to establish the potential of the contents of BISECT to produce novel antimicrobial natural products, by using a suite of tools to isolate strains most likely to have biosynthetic potential, such as those from the Actinomycetales order, and screen these strains under multiple conditions to increase the probability of bioactivity.

Despite numerous advances in the field of bioprospecting, traditional screening approaches continue to represent the dominant methodology that results in the discovery of novel compounds [32]. Successfully isolating novel producers is an essential part of reducing the risk of rediscovery and maximising the usefulness of samples from underexplored environments. A previous study by Esteves et al. [33] indicated that increasing media diversity represents an important strategy for increasing the diversity of cultured bacteria. Even the most effective sponge culturing studies (i.e., the most bacteria cultured in total) can only achieve ~10–14% recovery of species associated with a sponge sample [34], and given the extreme nature of the deep-sea environment we expected to achieve significantly less than this. While uncultured bacteria represent a promising avenue for novel opportunities, such as uncultured sponge symbiont *Entotheonella spp*. [35], culture-based approaches tend to select for bacteria with larger genomes, and larger genomes contain higher numbers of biosynthetic gene clusters [36]. For these reasons, there is a compelling rationale for focusing future efforts on optimising and diversifying the culture conditions employed during screening of the BISECT collection. However, it is clear that the method we chose for screening the isolates for bioactivity against pathogens (soft agar overlay assay) performed consistently, and allowed high throughput testing of hundreds of isolates. Once our assay protocols were refined and diversified we could show antibiotic activity for 13% of the strains cultured from sponge B01171. 

A previous study of sea-sponge-associated bacteria isolated strains from five phyla: Cyanobacteria, Actinobacteria, Bacteriodetes, Firmicutes and Proteobacteria [33]. This tallies with our findings, and given that all the sponge samples processed in this study were collected at a depth of >200 m the lack of photosynthetic Cyanobacteria is unsurprising. The increased numbers of Actinobacteria and reduction in Bacteroidetes between sponges B0789 and B0078 suggests that our use of different isolation media successfully encouraged the growth of bacterial species that are more likely to be producing bioactive compounds. No *Streptomyces* species were recovered which is intriguing given that this genus is usually well represented in culturing studies [37].

The actinomycetes genera recovered here have all been shown to demonstrate antimicrobial activity when previously isolated from sponge tissue in the literature [38]. The one exception to this is the *Chryseoglobus* genus, which at the time of writing has only one published species *Chryseoglobus frididaquae* [39]. Whilst no activity was identified during the screen, full genome analysis will enable further analysis of this rare deep-sea bacteria. *Micrococcus* and *Kocuria* dominated the overall diversity of the isolated actinomycetes, and these talented genera are already responsible for the most potent anti-MRSA compound identified from sponge associated bacteria. Kocurin is a thiazolyl peptide antibiotic which was found to be produced by *Micrococcus* and *Kocuria* inhabited sponges in the Florida Keys [31]. Notably, bioactive metabolites once thought to originate within the sponge have now been reclassifed as metabolites of symbiotic *Micrococcus* [40]. Overall rare marine actinomycetes, such as those isolated here, present an outstanding opportunity for future drug discovery [41].

Prior to OSMAC screens, we had identified only two unique species with antibacterial activity from 487 isolates: a *Micromonospora* species and several isolates belonging to the *Bacillus pumilus* clade that could not be distinguished by 16S rRNA gene sequencing [42]. Ninety bacterial strains that did not show activity under their original isolation conditions were selected from the BISECT collection for use in OSMAC screens. These included 57 unique strains (based on their 16S rRNA gene sequences) and 33 strains that were isolated on the actinomycete-targeting media R2A and M1 (16S rRNA genes were not sequenced). OSMAC screens of these 90 bacteria identified 22 unique strains with reproducible antibacterial activity—a hit rate for this sub-sample of 24%.

Bacteria related to some of the active strains identified in this study have been reported to have antibacterial activity. *Micromonospora* species are second only to *Streptomyces* as antibiotic producers [43,44]. *Bacillus* species have started to be recognised as rich sources of bioactive molecules [45]. As mentioned above, the novel peptide antibiotic kocurin was isolated from *Kocuria rhizophila* and *Micrococcus yunnanensis* [31]. A marine isolate related to *Erythrobacter citreus* was found to produce the cytotoxic natural product erythrazole B [46]. To the best of our knowledge, bioactive natural products are yet to be reported from the genera *Modestobacter* and *Stappia*, which could make our active isolates particularly promising as producers of novel compounds. We are now in the process of extracting and characterising putative bioactive compounds from these strains. 

The data presented herein hints at the abundance of novel microbially-derived chemistry present within BISECT. This study provides a description of the development of this resource and allied methodologies employed to date in the isolation and antimicrobial screening of bacterial strains from deep-sea sponges and serves to confirm how valuable culture-dependent approaches are when identifying marine-derived natural products. We hope that these data serve to nucleate interest and engagement from academic colleagues in the BISECT collection, and we commit to making its contents freely available to the academic community. Individuals interested in acquiring samples from BISECT are encouraged to contact the corresponding authors of this manuscript in the first instance. 

## 4. Materials and Methods 

### 4.1. Sponge Sample Collection

The sponge samples were collected as part of the Tracing Ocean Processes Using Corals and Sediments (TROPICS) research cruise in the equatorial Atlantic; from Tenerife to Trinidad aboard the RRS James Cook (expedition JC094), using the ROV Isis (13/10/13–30/11/13). There were five sampling locations from east to west: Carter and Knipovich seamounts in the eastern basin, the Vema fracture zone at the Mid-Atlantic Ridge and the Vayda and Gramberg seamounts in the western basin. 

The environmental conditions of the sites were recorded and varied as follows: temperature (2.8–9.9 °C), salinity (34.9–35.1 PSU), and oxygen molality (36.8–236.3 umol/kg). There are four classes of marine sponges—Calcarea, Demospongiae, Hexactinellida, and Homoscleromorpha—and all but the last class were collected. The sponges were photographed and subsampled in a controlled-temperature (4 °C) laboratory on board the ship before flash freezing them for storage at −80 °C.

### 4.2. Sponge Processing and Isolation of Bacterial Strains 

Sponge samples (1 g) were washed three times in 50 mL sterile artificial sea water (ASW; Crystal Sea Marine Mix, Marine Enterprise International, made to manufacturer’s instructions) for 30 s, with rocking. The samples were suspended in sterile artificial sea water (50 mL) and homogenised using a sterile pestle and mortar in a laminar flow hood to minimise environmental contamination. The sponge homogenate was then serially diluted (10^−1^–10^−4^) with sterile artificial sea water and spread onto a variety of agar types (Table S1). Duplicate plates were incubated at 28 °C and 4 °C for between two weeks and two months. Colonies were picked based on morphology and streaked onto fresh agar of the same type to grow as a monoculture in the same isolation temperature. The length of time each species took to grow varied. The strains were then directly stocked from the agar plate into Microorganism Preservation System Protect Cryotubes (Technical Service Consultants Ltd., Heylood, UK) and stored at −70 °C.

All media was made with ASW to encourage the growth of obligate marine bacteria and supplemented with cycloheximide (20 mg/mL) to inhibit the growth of fungi. For media targeting actinomycetes sodium pyruvate (1 mM) was also added to encourage their growth, and nalidixic acid (30 mg/mL) to inhibit the growth of Gram-negative bacteria.

### 4.3. Sponge Extract Preparation

A sponge extract used to supplement the growth media was prepared as follows. Sponge tissue (10 g) was homogenised in 30 mL phosphate buffered saline (PBS) over ice, then centrifuged (4700 rpm) for 10 min to remove large debris, the supernatant was aspirated and diluted to 45 mL with PBS. This was combined with 45 mL of methanol and then centrifuged (8500× *g*) for 10 min. The resulting supernatant was filtered through a 0.2 µM filter and stored at 4 °C.

### 4.4. Soft Agar Overlay Antimicrobial Activity Assay

Strains were grown as small patches until there was visual growth. *Staphylococcus aureus* Newman, *S. aureus* Mu50, *Pseudomonas aeruginosa* PA01, *Escherichia coli* BW55113, *Klebsiella pneumoniae* NCTC 5055 and *Acinetobacter baumannii* ATCC 19606 were grown in Mueller Hinton broth (Sigma-Aldrich), at 37 °C, for 16 h. Each culture was suspended at OD_600_ = 0.01 in warm (42 °C), soft Mueller Hinton agar (0.75% agar) and poured over the sponge bacterial isolates (10 mL/plate). The plates were incubated at 37 °C, for 16 h. A visible zone of inhibition surrounding the strain indicated the test strain had inhibited the growth of the pathogen.

### 4.5. 16S rRNA PCR Gene Amplification

Isolated colonies with distinct visual morphologies were re-streaked on their respective isolation media to ensure a monoculture. Species identification was performed using 16S rRNA gene amplification with universal bacterial primers 8F (5′ AGA GTT TGA TCC TGG CTC AG 3′) and 1512R (5′ ACG GCT ACC TTG TTA CGA CTT 3′) [47] and enabled amplification of near full length 16S rRNA gene fragment. Polymerase chain reaction (PCR) was conducted on bacterial genomic DNA using Platinum™ Hot Start PCR Master Mix (2X) (ThermoFisher) or REDTaq^®^ ReadyMix™ PCR Reaction Mix (Sigma-Aldrich, St. Louis, MO, USA). The following cycle was used: heating to 94 °C for 10 min, then 35 cycles of denaturation (94 °C, 1 min), annealing (56 °C, 1 min) and extension (72 °C, 1.5 min), then a final elongation (72 °C, 10 min). The 16S rRNA gene product size (~1450 bp) was confirmed using gel electrophoresis, then excised using the QIAquick Gel Extraction Kit (Qiagen) or directly purified using the QIAquick PCR Purification Kit (Qiagen) according to the manufacturer’s instructions. Amplicons were then Sanger sequenced (Eurofins Genomics) and the species identified using BLAST and the 16S ribosomal database. 

### 4.6. Phylogenetic Analysis

Combined 16S rRNA sequences for Actinobacteria were used to construct a neighbour-joining tree. The phylogenetic tree was constructed with the Tamura–Nei model with a discrete gamma distribution using MEGAX (10.1.8) (Mega Limited, Auckland, New Zealand) [48]. The constructed tree was evaluated through bootstrap analysis performed 1000 times. 

### 4.7. OSMAC Screening

Ninety bacterial isolates were assigned to a number and position on duplicate 45-membered grids on a 120 mm Petri dish. Each isolate was spotted from its frozen glycerol stock onto an agar plate at its assigned position in the grid. The plates were left to dry and incubated at 28 °C for seven days. Plates were overlaid with *S. aureus* Newman or *A. baumannii* suspended in soft agar (as per Section 4.4). The plates were then examined for clear zones where the growth of the pathogen was inhibited. When more than one sponge isolate fell within a zone of inhibition they were all considered to be ‘hits’. To verify the activities of these ‘hits’, each one was grown axenically on the same OSMAC medium at 28 °C for seven days and tested for activity using the soft agar overlay assay as above. This assay was repeated in triplicate, and the ‘hits’ were classified as ‘hit again’, ‘no activity’ or ‘no growth’. For the ‘hit again’ isolates genomic DNA was extracted and the 16S rRNA genes were amplified using PCR and then sequenced to classify the isolates (see Section 4.5).

Two OSMAC screens were designed. The first screen used ISP2 (Supplementary Table S1) as a complex base medium with variation in physical, chemical, and biological factors (Supplementary Table S5). Colloidal chitin was prepared following the protocol used by Giubergia et al. 2016 [49], and an aqueous sponge extract was prepared following the protocol used by Selvin et al. [50]. A ‘lysed pathogens solution’ was prepared by collecting 5 µL each of freshly streaked colonies of *E. coli* BW25113, *S. aureus* Newman, *S. aureus* Mu50, *K. pneumoniae* NCTC 5055, *A. baumannii* ATCC 19606, *P. aeruginosa* PAO1, *E. faecalis* UB591, and *B. subtilis* and combining them together in 1 mL of PBS, and heating the solution at 95 °C for 30 min. The sterility of the solution was verified by plating it on Mueller–Hinton agar and checking for bacterial growth after incubation at 37 °C for 16 h. The second OSMAC screen used M9 as a minimal base medium (Supplementary Table S6) and additional carbon sources (colloidal chitin, dextrose, glycerol, mannitol, soluble starch and succinate) were added. M9 without the addition of a carbon source was also used.

## 5. Conclusions

Here we report our first steps in characterising a new collection of deep-sea bacteria (BISECT) and in understanding the biosynthetic potential of these isolates. Our initial focus is on antibiotic discovery, prompted by the emerging global health crisis of antimicrobial resistance. In agreement with previous studies, we show that a variety of deep-sea bacteria can be cultured under laboratory conditions, and that these isolates should be grown on diverse media to promote the broadest range of natural products. While further work is required to fully exploit this collection, our results are an encouraging platform for further discovery.

## Figures and Tables

**Table 1:** Sponge samples used in this study. *Sample ID* is the in-house nomenclature.

Order of Processing	Sample ID	Latitude (N)	Longitude (W)	Depth (m)	Sampling Region	Visual ID	Total Bacterial Isolates ^a^
1	B01607	10°96′49′′	44°36′58′′	2887	Vema fracture zone	Hexactinellid	37
2	B01641	14°53′3′′	48°7′27′′	868	Vayda seamount	Hexactinellid	55
3	B01661	14°51′47′′	48°14′29′′	1483	Vayda seamount	Demosponge	73
4	B0789	14°53′25′′	48°9′7′′	824	Vayda seamount	Hexactinellid	136
5	B0078	5°37′30′′	26°57′29′′	971	Knipovich seamount	Demosponge	182
6	B01171	5°35′43′′	25°58′28′′	2257	Knipovich seamount	Demosponge	229

^a^ As the project progressed, sponges were processed with an increasing diversity of media conditions, thus the numbers of total bacterial isolates are not directly comparable between samples.

**Table 2:** Conditions used to culture bacteria from sponge samples. See Table S1 for agar constituents.

Sponge Sample	Agar Type	Colonies Isolated	
		28 °C	4 °C
B01607	AIA-XO	0	0
	Marine	19	19
	Peptone-starch	0	0
	TOTAL	19	19
B01641	AIA-XO	17	0
	Marine	30	9
	Peptone-starch	1	0
	TOTAL	48	9
B01661	AIA-XO	0	0
	Marine	52	20
	Peptone-starch	8	0
	TOTAL	60	20
B0789	60:40	2	1
	AIA	16	11
	AIA-XO	4	-
	Basic	7	4
	Marine	43	21
	Peptone-starch	10	18
	TOTAL	82	55
B0078	60:40	10	0
	ISP2	50	0
	M1	25	1
	Marine	23	4
	Peptone-starch	11	0
	R2A	27	4
	SCA	27	0
	TOTAL	173	9
B01171	60/40	7	3
	ISP2	17	5
	ISP2/10	7	4
	ISP4	4	3
	M1	5	2
	MB1/2	11	10
	MB20	9	10
	MB20-NO_3_	3	-
	Mucin	7	13
	Peptone-starch	22	7
	R2A	3	5
	SA1	8	0
	SAC	12	0
	Spicule-extract	12	8
	TOTAL	127	70

## Supplementary Materials

The following are available online at https://www.mdpi.com/2079-6382/9/8/509/s1, **Table S1.** Agar composition; **Table S2.** Pathogenic bacterial strains used in this study; **Table S3.** Activity of ‘hit’ strains from B01171 using a soft agar overlay assay; **Table S4.** Closest relatives and activities of the 22 OSMAC hits from the first screen on ISP2 media; **Table S5.** ISP2 media for first OSMAC screen; **Table S6.** M9 media for second OSMAC screen, and supplementary references.
